# SARS-CoV-2 NSP12 associates with TRiC and the P323L substitution acts as a host adaption

**DOI:** 10.1128/jvi.00424-23

**Published:** 2023-11-06

**Authors:** Muhannad Alruwaili, Stuart Armstrong, Tessa Prince, Maximillian Erdmann, David A. Matthews, Lisa Luu, Andrew Davidson, Waleed Aljabr, Julian A. Hiscox

**Affiliations:** 1 Institute of Infection, Veterinary and Ecological Sciences, University of Liverpool, Liverpool, United Kingdom; 2 Medical Laboratory Technology Department, Northern Border University, Arar City, Saudi Arabia; 3 School of Cellular and Molecular Medicine, University of Bristol, Bristol, United Kingdom; 4 King Fahad Medical City, Riyadh, Saudi Arabia; 5 A*STAR Infectious Diseases Laboratories (A*STAR ID Labs), Agency for Science, Technology and Research (A*STAR), Singapore; The Peter Doherty Institute for Infection and Immunity, Melbourne, Victoria, Australia

**Keywords:** SARS-CoV-2, NSP12, P323L, TRiC, phosphatase

## Abstract

SARS-CoV-2 emerged in the human population in late 2019, and human-to-human transmission has dominated the evolutionary landscape and driven the selection of different lineages. The first major change that resulted in increased transmission was the D614G substitution in the spike protein. This was accompanied by the P323L substitution in the viral RNA-dependent RNA polymerase (NSP12). Together with D614G, these changes are the root of the predominant global SARS-CoV-2 landscape. Here, we found that NSP12 formed an interactome with cellular proteins. The functioning of NSP12 was dependent on the T-complex protein ring complex, a molecular chaperone. In contrast, there was a differential association between NSP12 variants and components of a phosphatase complex (PP2/PP2A and STRN3). The virus expressing NSP12_L323_ was less sensitive to perturbations in PP2A and supports the paradigm that ongoing genotype to phenotype adaptation of SARS-CoV-2 in humans is not exclusively restricted to the spike protein.

**IMPORTANCE:**

SARS-CoV-2 has caused a worldwide health and economic crisis. During the course of the pandemic, genetic changes occurred in the virus, which have resulted in new properties of the virus—particularly around gains in transmission and the ability to partially evade either natural or vaccine-acquired immunity. Some of these viruses have been labeled Variants of Concern (VoCs). At the root of all VoCs are two mutations, one in the viral spike protein that has been very well characterized and the other in the virus polymerase (NSP12). This is the viral protein responsible for replicating the genome. We show that NSP12 associates with host cell proteins that act as a scaffold to facilitate the function of this protein. Furthermore, we found that different variants of NSP12 interact with host cell proteins in subtle and different ways, which affect function.

## INTRODUCTION

The first major changes in the SARS-CoV-2 genome in the human population after spillovers from the intermediate animal host in the Huanan Seafood Wholesale Market (1, 2) were the D614G substitution in the spike glycoprotein (spike_D614G_) accompanied by the P323L substitution in the viral RNA-dependent RNA polymerase (NSP12) (NSP12_P323L_). These changes were associated with increased transmissibility and fitness (3) and mechanistically were linked to the spike_D614G_ substitution resulting in increased cell entry (4). While the spike_D614G_ substitution occurred in several different lineages, including lineage A, the combination of spike_D614G_ and NSP12_P323L_ substitutions was associated with the emergence of the B.1 lineage (5) and descendent viruses that have come to dominate the global genomic landscape of SARS-CoV-2 (6 – 8). Focus on these changes was placed on spike_D614G_, with the NSP12_P323L_ substitution being considered a potentially fortuitous hitchhiker (9). However, only the double mutation was epidemiologically successful, and variants with single mutations, spike_D614_ and NSP12_P323_, were a fraction of the global landscape (10). In support of NSP12_P323L_ being biologically relevant, in animal models, viruses with NSP12_L323_ had greater fitness than viruses with NSP12_P323_, suggesting a functional role in virus biology (11).

The role of the NSP12_P323L_ substitution in SARS-CoV-2 biology has not been elucidated. In the virus cellular life cycle, NSP12 is the catalytic subunit of the polymerase complex. As such, the protein does not function in isolation and forms a complex with (at least) two other viral proteins including the co-factors NSP7 and NSP8 (12, 13). In a wider context, the replication complex functions with other viral proteins that alter the structure of the intracellular membranes (14). The SARS-CoV-2 (and SARS-CoV) NSP12 is divided into several domains including the N-terminal nucleotidyl transferase (NiRAN) domain, the interface between amino acids 251 and 398, and the C-terminal polymerase domain. Structural analysis suggested that the interface could act as a protein interaction junction linking NiRAN with the fingers of the polymerase domain and NSP8 (13). The NSP12_P323L_ substitution lies within the interface domain and is not obvious from structural analysis how this might alter the function of the protein.

We hypothesized that the NSP12_P323L_ substitution was a host adaptation that altered interaction with host cell proteins and functioned in virus RNA synthesis. Host proteins have been shown to interact with SARS-CoV-2 proteins (15), and amino acid substitutions within these viral proteins may modulate their interaction with host proteins and function in the viral life cycle. Several RNA-dependent RNA polymerases (RdRps) from different viruses with RNA genomes interact with host proteins, including chaperones to promote stability and co-factors involved in replication (16 – 18).

To investigate this for SARS-CoV-2 NSP12, biochemical and virological approaches were combined to functionally characterize the interaction between NSP12_P323_ and NSP12_L323_ and the host cell proteome. The T-complex protein ring complex (TRiC) was identified as binding to both variants of NSP12 and disrupting chaperone activity ablated virus biology. In contrast, interactome analysis showed that protein phosphatase 2 (PP2/PP2A) associated more with NSP12_L323_ rather than NSP12_P323_. Reduction in the abundance of PP2A in virus-infected cells resulted in a concomitant change in viral RNA synthesis but the virus expressing NSP12_L323_ was less sensitive to these effects than the virus expressing NSP12_P323_.

## RESULTS

### Both the NSP12_P323L_ variants interact with TRiC but PP2A and STRN3 favor interaction with NSP12_L323_

**Fig 1:**
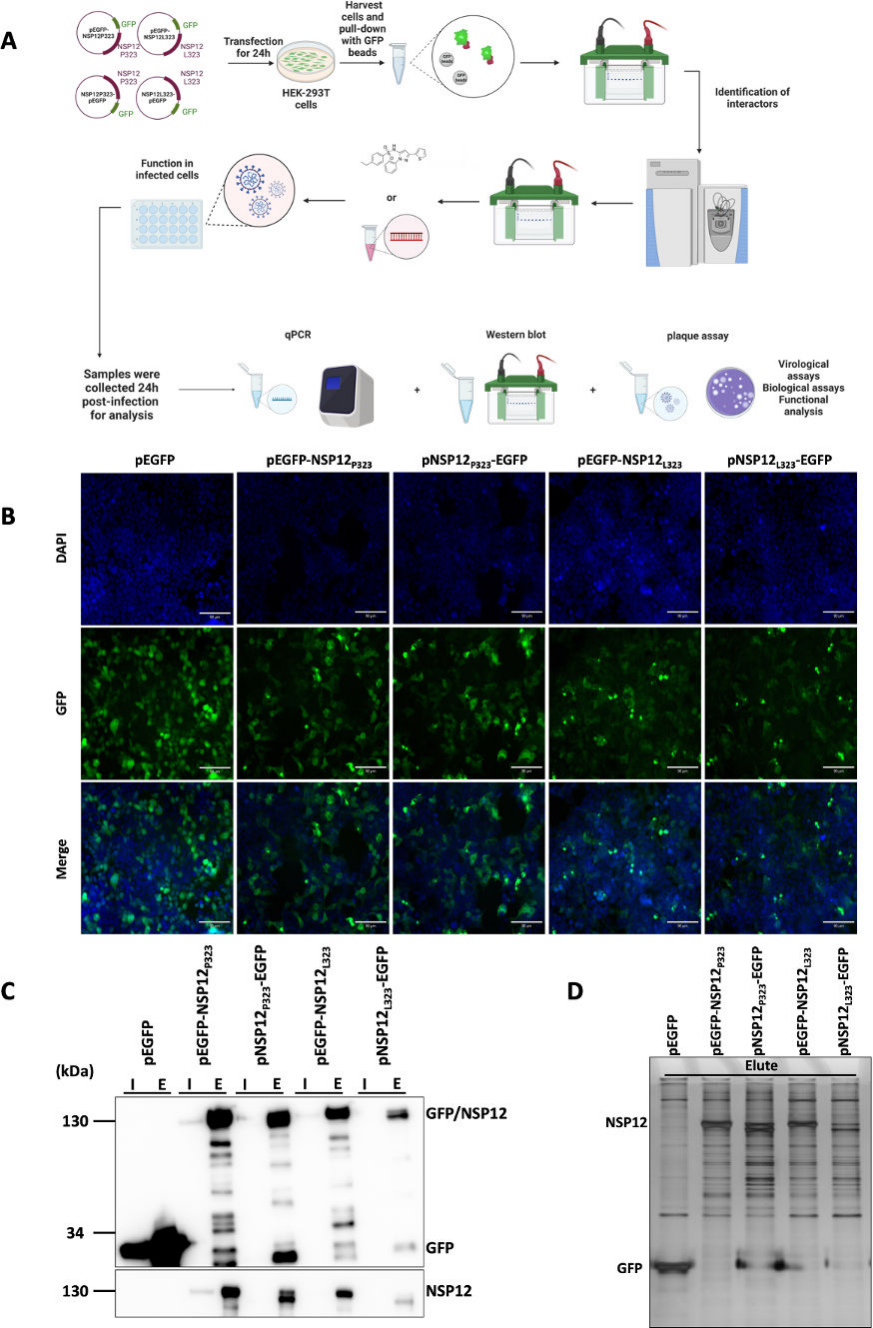
Expression of EGFP, EGFP-NSP12_P323_, NSP12_P323_-EGFP, EGFP-NSP12_L323_, and NSP12_L323_-EGFP in HEK-293T cells. (**A**) Schematic diagram of the general methodology and approach used in this study. The NSP12 fusion proteins were expressed in HEK-293T cells and immunoprecipitated using a GFP-Trap. LC-MS/MS was used to identify the interactome, and western blot was used to validate key interactions. The functional implications of these findings were investigated using inhibitors or ablating the protein/function of interest, and the effect on viral biology was quantified. (**B**) Expression of EGFP, EGFP-NSP12_P323_, NSP12_P323_-EGFP, EGFP-NSP12_L323_, and NSP12_L323_-EGFP in HEK-293T cells was confirmed by immunofluorescence microscopy with DAPI (blue) staining the nucleus. Scale bar, 90 μM. (**C**) Immunoblot analysis of input (I) and elute (E) EGFP, EGFP-NSP12_P323_, NSP12_P323_-EGFP, EGFP-NSP12_L323_, and NSP12_L323_-EGFP by western blot. (D) Eluted samples for the mass spectrometry analysis were run on SDS-PAGE and stained with a silver stain, showing a difference in protein profile.

To investigate the protein-protein interactions formed by NSP12 and whether there was a difference between NSP12_P323_ and NSP12_L323_, an enhanced green fluorescent protein (EGFP)-based pull-down approach was used in conjunction with mass spectrometry (MS) to identify and quantify potential cellular interactors, followed by functional analysis for relevance to the virus life cycle (Fig. 1A). Previously, we have used this approach to define the cellular interactome of a variety of different viral proteins, including the human respiratory syncytial virus RNA-dependent RNA polymerase (L protein) (16), polymerase co-factors for Ebola virus (19), and the avian coronavirus nucleoprotein (20). Many of these viral proteins retained biological activity with the EGFP tag. Here, either variant of NSP12 was fused to EGFP at either the C or N terminal and expressed in human cells in culture under the control of a CMV promoter (Fig. 1A). The placing of the EGFP tag at either N-terminal or C-terminal was to allow for any steric hindrance imparted by the EGFP moiety, which might have affected protein-protein interactions. The plasmid constructs (p) generated were pEGFP-NSP12_P323_, pNSP12_P323_-EGFP, pEGFP-NSP12_L323_, and pNSP12_L323_-EGFP. These plasmids were separately transfected into human embryonic kidney 293 (HEK-293T) cells, and expression was confirmed by fluorescence, western blot, and silver staining and compared to cells transfected with pEGFP only (expressing EGFP) (Fig. 1B and C, respectively). This indicated that all four fusion proteins were expressed in cells (higher resolution imaging of each expression construct is shown in Fig. S1). Western blot analysis using antibodies to either EGFP or NSP12 confirmed specific expression of the NSP12 moiety (Fig. 1C), with overexposure suggesting the presence of breakdown/cleaved products. Silver stain analysis of protein lysates from pulldowns from the transfected cells highlighted the presence of the EGFP control and the NSP12 fusion proteins, with other protein species being present. These latter are suggestive of NSP12-interacting partners, given the difference to EGFP only (Fig. 1D).

**Fig 2:**
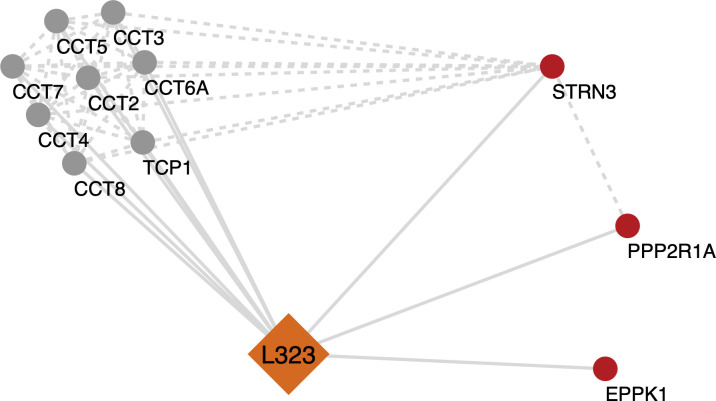
Analysis of selected significant cellular proteins that potentially interact with NSP12_L323_. Potential protein-protein interaction networks of NSP12_L323_ were derived using Cytoscape from the mass spectrometry interactome data. Multiple proteins were found to be associated with NSP12_L323_. In this simplified figure, the highly enriched chaperonin-containing TCP1 (TRiC/CCT complex) is involved in the folding and assembly of proteins associated with the NSP12_L323_ interactome. A few proteins were identified to be enhanced in the NSP12_L323_ interactome (STRN3 and PP2R1A, which are part of the PP2A phosphatase family). Full interactome data for NSP12_P323_ and NSP12_L323_ are presented in Fig. S1–S4 and Tables S1 to S4.

To identify and quantify potential differences between the cellular interactomes of NSP12_P323_ or NSP12_L323_, the five expression constructs were transfected five separate times into HEK-293T cells. Cells were lysed, and the NSP12 moieties with EGFP and associated complexes were immunoprecipitated using an EGFP-trap. Each pull-down complex was characterized by mass spectrometry, and cellular proteins were compared across the different expression constructs. Criteria for inclusion as a positive “hit” included identification of a protein by two or more unique peptides, scoring for each identified protein with a SAINT (Significance Analysis of INTeractome) (21) Bayesian false-discovery rate (BFDR) equal to or below 0.05. A MiST (Mass spectrometry interaction STatistics) (22) score of 0.6 and above was used as an additional filter to identify proteins unique to a particular construct. After this filtering process (BFDR ≤ 0.05), 91, 40, 97, and 50 proteins were identified as interacting with EGFP-NSP12_P323_, NSP12_P323_-EGFP, EGFP-NSP12_L323_, and NSP12_L323_-EGFP, respectively (Tables S1–S4). The data indicated several common interactions across the four target proteins. These included components of the TRiC [or also known as chaperonin-containing TCP-1 (CCT)], which is a multiple protein complex acting as a protein chaperone. However, some interactions were more enhanced/gained in EGFP-NSP12_L323_ and NSP12_L323_-EGFP compared to EGFP-NSP12_P323_ and NSP12_P323_-EGFP. This included proteins serine/threonine-protein phosphatase 2A 65 kDa regulatory subunit A alpha isoform [PPP2R1A, a subunit of protein phosphatase 2 (PP2), also known as PP2A] and striatin-3 (STRN3) and epiplakin (EPPK1) (Fig. 2) (see Fig. S2 to S5 for interactome maps of the individual expression proteins). STRN3 can act as a regulatory subunit of PP2A (23). In cells, the function of PP2A is regulating phosphorylation and opposing the action of cellular kinases.

### Validation of TRiC and PP2A and STRN3 interactions with NSP12_P323_ and NSP12_L323_

**Fig 3:**
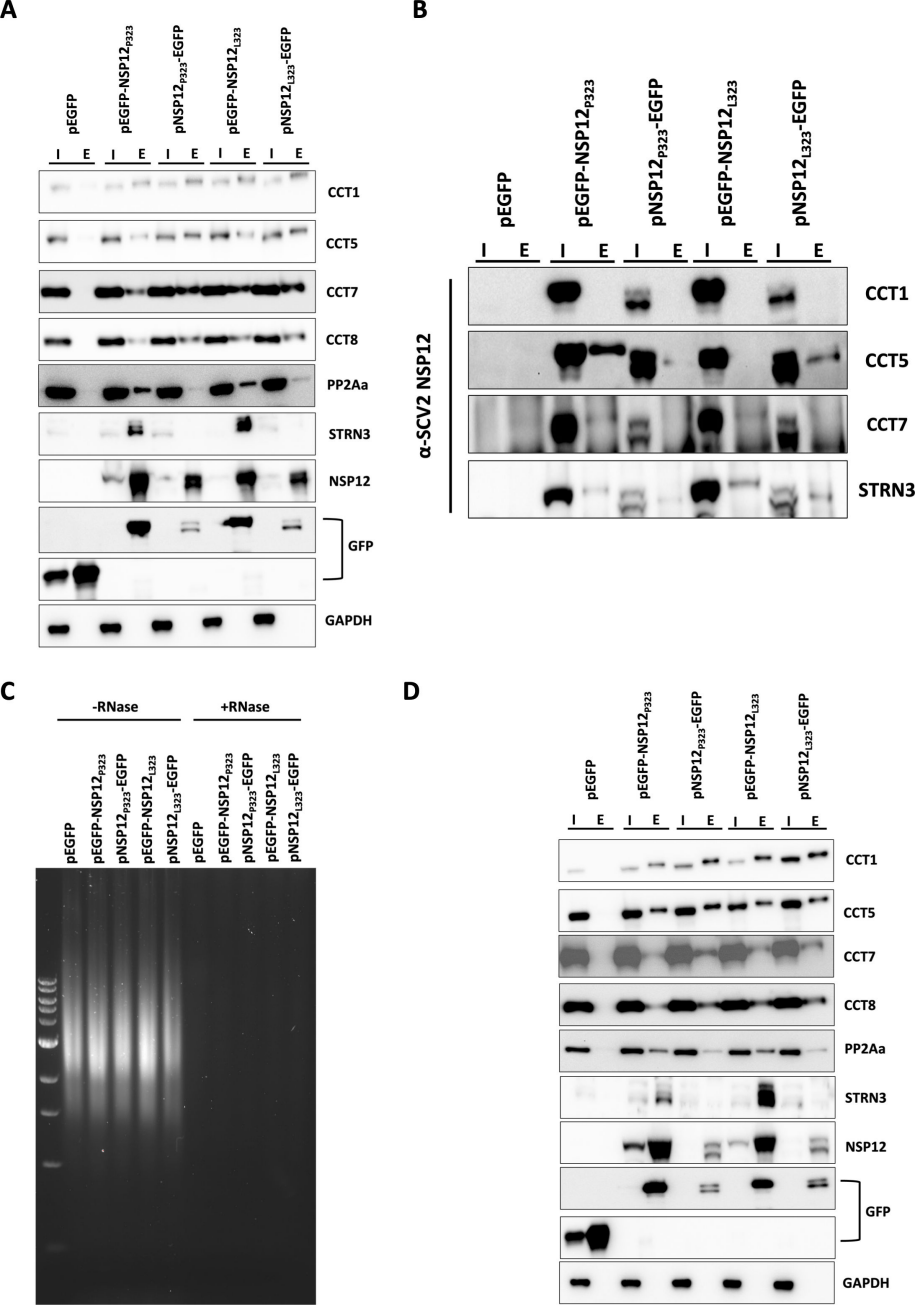
Validation of protein-protein interactions between EGFP tagged NSP12_P323_ or NSP12_L323_ and the host proteins in HEK-293T cells. (**A**) Transfection and pulldowns were repeated with EGFP, EGFP tagged NSP12_P323_, and NSP12_L323_ at the C- and N-terminus for the detection of CCT1, CCT5, CCT7, CCT8, PP2Aa, and STRN3 (I = input, E = elute). (**B**) Reverse pulldowns for CCT1, CCT5, CCT7, and STRN3 and detection of EGFP, EGFP-NSP12_P323_, NSP12_P323_-EGFP, EGFP-NSP12_L323_, and NSP12_L323_-EGFP with a specific antibody against SARS-CoV-2 (SCV2) NSP12. (**C**) Agarose gel electrophoresis showing that the treatment with RNase (+RNase) removed host RNA from cell lysate compared to untreated (−RNase). (**D**) Samples from panel (**C**) were run on SDS-PAGE for the detection of host proteins in the absence of RNA using a forward pull-down approach.

The interactome analysis indicated that NSP12_L323_ interacted with the TRiC complex, which itself was linked to PP2A and STRN3, and these were subject to further investigation. Orthogonal techniques were used to validate the potential interaction between these cellular proteins and the viral targets. The transfection/expression experiments were repeated for each NSP12/EGFP fusion protein using both forward (Fig. 3A) and reverse pulldowns (Fig. 3B) with specific antibodies to selected components of the TRiC —CCT1, CCT5, CCT7, and CCT8, as well as antibodies to PP2A and STRN3. The forward and reverse pulldowns confirmed the findings of the mass spectrometry analysis. A comparison of the cellular interactors of both variants of NSP12 showed that PP2A and STRN3 were more specific to EGFP-tagged NSP12_L323_ than EGFP-tagged NSP12_P323_. To investigate whether these interactions were mediated through binding to RNA (which may be plausible given NSP12 can associate with RNA), forward pulldowns were treated with RNase to remove species of cellular RNA (Fig. 3C). Analysis of these treated pulldowns with the specific antibodies to TRiC, PP2A, and STRN3 indicated that none were mediated by RNA (Fig. 3D).

### Functional role of TRiC in the life cycle of SARS-CoV-2

**Fig 4:**
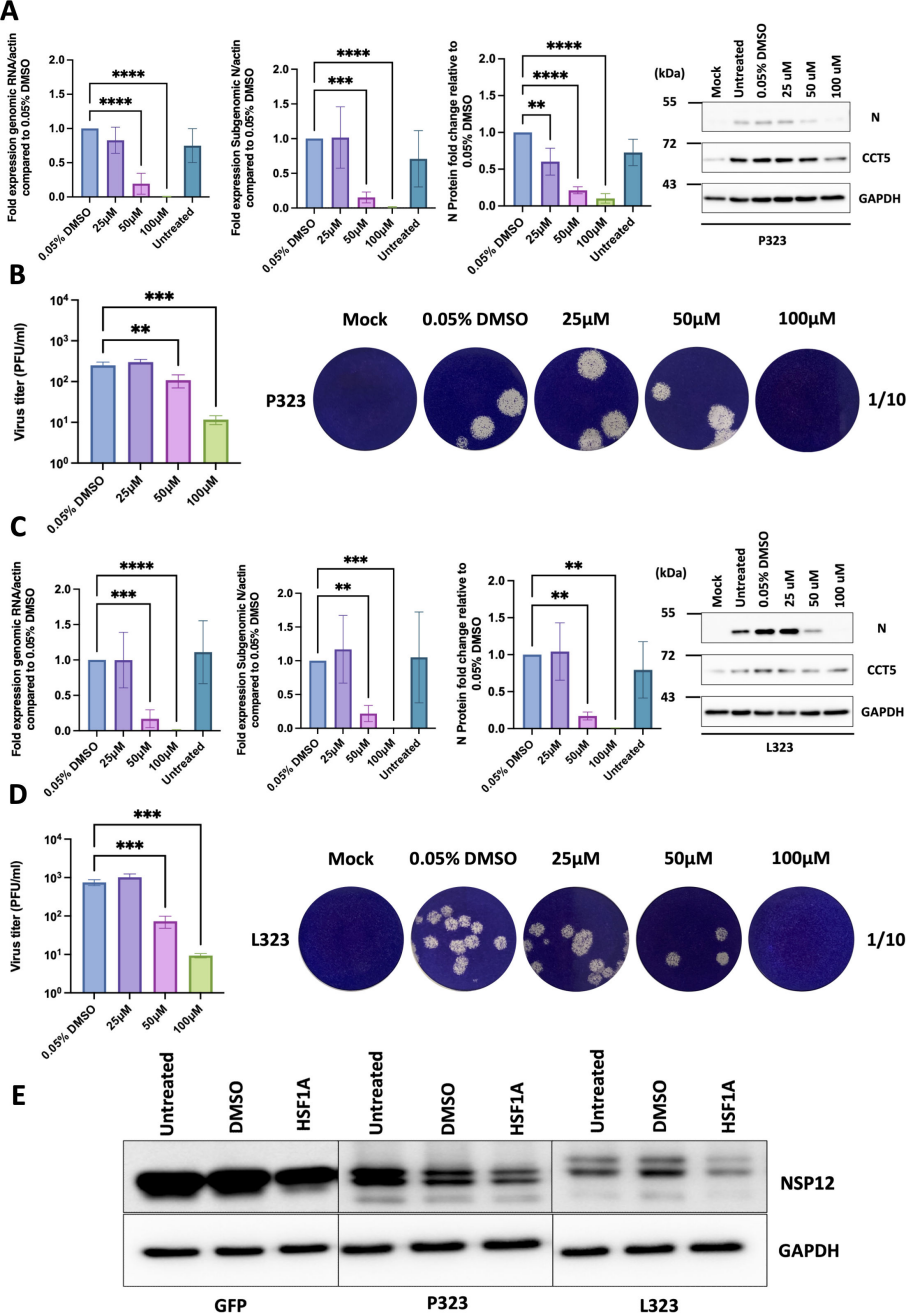
Determining the effect of TRiC/CCT inhibitor HSF1A on the recombinant virus P323 and L323 replication. (**A and C**) ACE2-A549 cells were pre-treated with either DMSO (control) or HSF1A (25, 50, or 100 µM) for 4 hours before being mock-infected or infected with recombinant viruses expressing either NSP12_P323_ or NSP12_L323_ viruses (MOI of 0.1) and then maintained in DMSO or HSF1A. At 24 hours after infection, gRNA and sgRNA levels were measured by qRT-PCR and normalized to β-actin. At the same time, whole cell lysate samples were collected, and the expression of nucleocapsid and CCT proteins was detected using western blot analysis with specific antibodies. GAPDH was used as a loading control. ImageJ was used to assess the expression of the nucleocapsid protein. Viral protein expressions were normalized to GAPDH. (**B and D**) ACE2-A549 cells were pre-treated for 4 hours with DMSO (control) or for 4 hours with HSF1A (25, 50, and 100 µM) before being mock-infected or infected with reverse genetically created P323 or L323 viruses at an MOI of 0.1. Infected cells were maintained in media containing 0.05% DMSO or the previous concentrations of HSF1A for 24 hours. Viral titers were determined by plaque assay. For the plaque assays, representative image data shown are from one experiment. (**E**) HEK-293T cells were transfected with a plasmid containing EGFP as a control, NSP12_P323_-EGFP, or NSP12_L323_-EGFP, respectively. Cells were then treated at 4 hours post-transfection with either DMSO (0.05%) or HSF1A (50 µM). Whole cell lysates were collected, and western blot analysis was performed to detect the abundance of EGFP, NSP12_P323_-EGFP, and NSP12_L323_-EGFP proteins. GAPDH was used as a loading control. **P < 0.01, ***P < 0.001, and ****P < 0.0001 by one-way ANOVA with Dunnett’s multiple comparison test. All error bars represent the standard deviation. Data shown in panels **A–D** represent the mean of three independent experiments.

To investigate whether the interaction between the TRiC and NSP12 was important for SARS-CoV-2 replication, the function of TRiC was inhibited using the small molecule inhibitor HSF1A (heat shock transcription factor 1). This inhibitor was used because it has been reported to bind to TriC subunits and inhibit the activity of the complex without affecting the hydrolysis of ATP (24). A dose-dependent assay was used to determine what concentration of the inhibitor could be used without affecting cell viability in either angiotensin-converting enzyme 2 (ACE2-A549) cells (used in infection assays—Fig. S6A) or HEK-293T cells (used in overexpression analysis—Fig. S6B). The cell viability in the presence of different concentrations of HSF1A was similar between the two cell types, with 200 μM being equivalent to that induced by etoposide (which is a known agent/positive control that induces apoptosis). We hypothesized that disruption of TRiC would have a negative impact on SARS-CoV-2 biology and disrupt NSP12. Therefore, to test this hypothesis, the functioning of TRiC was disrupted with 25, 50, and 100 μM HSF1A in ACE2-A549 cells. These cells were infected with either recombinant SARS-CoV-2 expressing NSP12_P323_ or recombinant SARS-CoV-2 expressing NSP12_L323_. The effect of no inhibitor and inhibitor on virus biology was assessed. Several measures were used including RT-qPCR to determine the abundance of genomic and subgenomic RNA, the abundance of the nucleoprotein by western blot, and comparison of viral titers. The data indicated that inhibitionTRiC affected the biology of both SARS-CoV-2 expressing NSP12_P323_ (Fig. 4A and B) and SARS-CoV-2 expressing NSP12_L323_ (Fig. 4C and D). This resulted in a significant reduction in the expression of viral RNAs, viral protein, and viral titers compared to virus grown in cells that were unexposed or received the vehicle-only control (DMSO).

The interaction between protein chaperones and viral-encoded RdRps suggested that these complexes were involved in maintaining/facilitating the stability and function of the viral protein (e.g., references 16 and 17). Given TRiC is involved in protein folding and stability, we hypothesized that inhibition of TRiC would result in the disruption of NSP12 and manifest in decreased abundance of the protein. To investigate this, NSP12_P323_-EGFP or NSP12_L323_-EGFP were overexpressed in HEK-293T cells exposed to HSF1A. The data indicated that in the presence of HSF1A, NSP12_P323_-EGFP and NSP12_L323_-EGFP were less abundant compared to the expression of these proteins in cells exposed to vehicle-only control (DMSO) or in the absence of vehicle/vehicle and inhibitor (Fig. 4E). Taken together, the data suggested that the inhibitor disrupted the activity of TRiC and that this structure was important in maintaining the stability of NSP12.

### The NSP12_P323L_ substitution may act as a host adaptation mutation

The cellular interactomes of EGFP-NSP12_P323_ or EGFP-NSP12_L323_ were identical in terms of interactions with cellular proteins (TRiC being an example) but differed in the degree of potential association with PP2Aa and STRN3. Mass spectrometry analysis indicated that these latter two proteins were more abundant in pulldowns where EGFP-NSP12_L323_ was used as a bait compared to EGFP-NSP12_P323_. To investigate whether these interactions had biological relevance, the mRNAs encoding STRN3 and PP2Aa were ablated using an siRNA-based approach (Fig. S7A and B, respectively), which resulted in a decrease in the abundance of both proteins after 48 hours of exposure (Fig. S7C and D, respectively) with little impact on cell viability (Fig. S7E and F for STRN3 and PP2Aa, respectively). In both depletions, a non-targeting siRNA and siRNA to GAPDH were used as a positive control to monitor knockdown and assess potential off-target effects.

**Fig 5:**
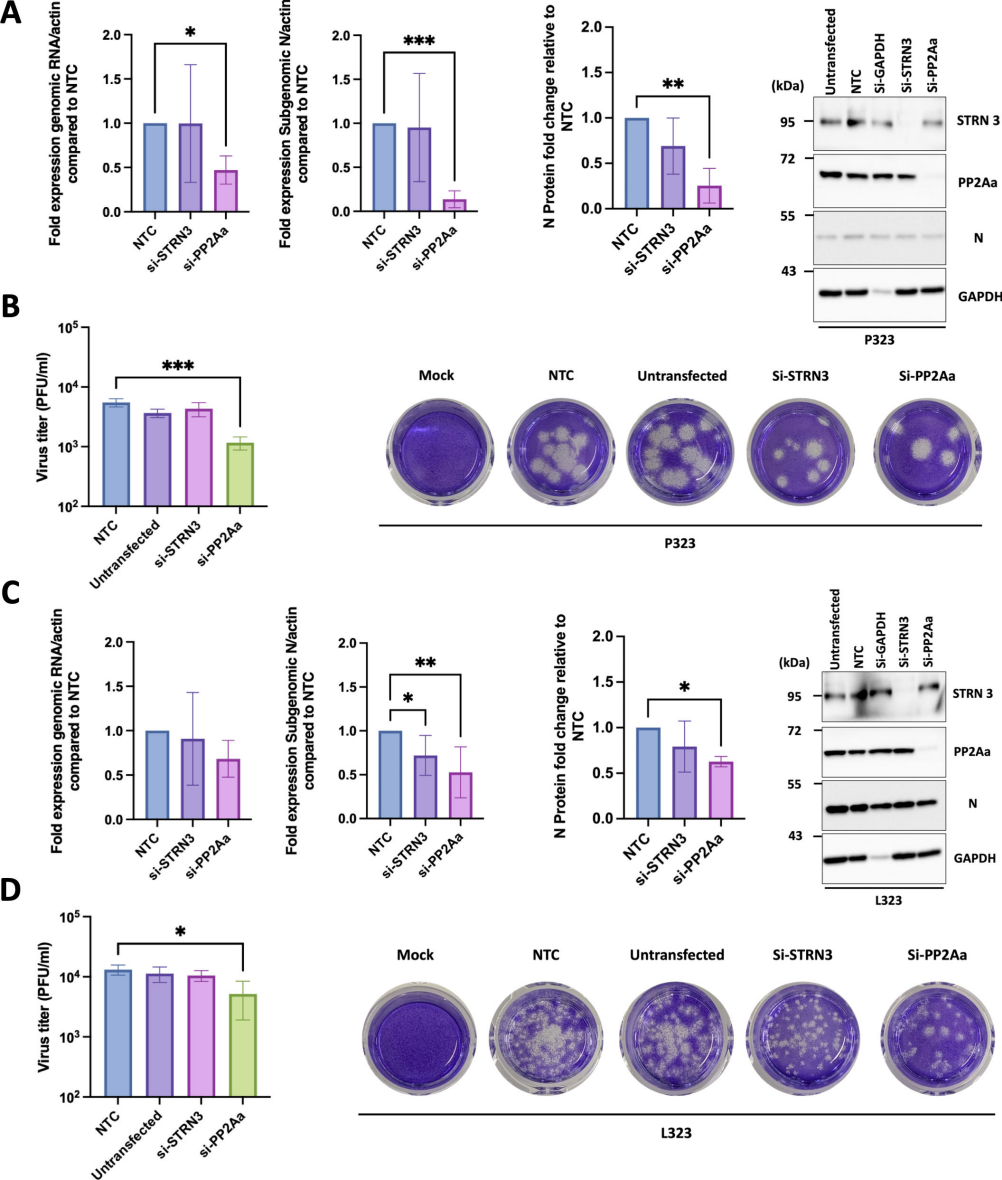
The impact of STRN3 and PP2Aa suppression on the replication of recombinant P323 and L323 viruses. (**A and C**) ACE2-A549 cells were mock-transfected or transfected with non-targeting siRNA (NTC), si-GAPDH (to monitor transfection efficacy), si-STRN3, or si-PP2Aa for 48 hours. Subsequently, the ACE2-A549 cells were infected with recombinant viruses expressing either NSP12_P323_ or NSP12_L323_ (MOI = 1 for 24 hours). RNA samples were extracted, and gRNA and sgRNA levels of the recombinant viruses were determined by qRT-PCR and normalized to β-actin. At the same time, whole cell lysate samples were collected, and the expression of nucleocapsid, STRN, and PP2Aa proteins was detected using western blot analysis. GAPDH was used as a loading control. ImageJ was used to assess the expression of the nucleocapsid protein. Viral protein expression was normalized to GAPDH. (**B and D**) Supernatants, from the experiments described in panels A and C, were collected and viral titers were determined by plaque assay. *P < 0.05, **P < 0.01, and ***P < 0.001 by one-way ANOVA with Dunnett’s multiple comparison test. Data shown in panels **A–D** represent the mean of three independent experiments. All error bars represent the standard deviation. For the plaque assays, representative image data shown are from one experiment.

For the infection experiments, ACE2-A549 cells were depleted of STRN3 and PP2Aa for 48 hours prior to infection with the recombinant viruses expressing either NSP12_P323_ or NSP12_L323_. Cells were infected for 24 hours, and viral biology was assayed using RT-qPCR to detect and compare viral RNA, and western blot to identify the nucleoprotein, STRN3, and PP2Aa (Fig. 5). The data indicated that depletion of STRN3 in cells infected with SARS-CoV-2 NSP12_P323_ resulted in no significant change in the abundance of viral RNAs, proteins, or titer (Fig. 5A and B). In contrast, for cells infected with SARS-CoV-2 NSP12_P323_ and depleted of PP2A, there was a significant reduction in the abundance of viral RNAs, proteins, and titer (Fig. 5A and B). In cells infected with SARS-CoV-2 NSP12_L323_, a slightly different pattern emerged. In cells depleted of STRN3 (Fig. 5C), there was only a reduction in the abundance of the nucleocapsid protein sgRNA, the other virological factors were not significantly different. For the depletion of PP2A in cells infected with SARS-CoV-2 NSP12_L323_, there was a significant reduction in the abundance of viral subgenomic mRNA (but the abundance of genomic RNA remained unchanged), nucleocapsid protein, and viral titer. The major difference between SARS-CoV-2 NSP12_P323_ and SARS-CoV-2 NSP12_L323_ was that the latter variant was less sensitive to the depletion of PP2Aa than the former.

## DISCUSSION

SARS-CoV-2 emerged into the human population in late 2019 after several zoonotic jumps (1, 2) and presumably transiting through population bottlenecks. The first major genomic change that resulted in different viral phenotypes was spike_D614G_ glycoprotein accompanied by NSP12_P323L_. Several studies demonstrated that the spike_D614G_ substitution resulted in a gain in viral fitness including increased infectivity and stability of virions resulting in greater viral loads in the upper respiratory tract of people infected with SARS-CoV-2 (compared to contemporary viruses at the time) (3). The NSP12_P323L_ substitution was suggested to have occurred concurrently (10). Analysis of SARS-CoV-2 genomes in the containment and first lock down phase in the UK in 2020 showed rapid emergence of spike_D614G_ and NSP12_P323L_ (11). Infection experiments using *in vivo* models for COVID-19 suggested that the NSP12_P323L_ substitution also contributed a fitness advantage—over and above that provided by spike_D614G_ (11).

While substitutions within SARS-CoV-2 may act at a structural level to stabilize RNA structure (25) or viral proteins such as NSP12 (26), these may also facilitate protein-protein interactions both between viral proteins and with host proteins. Several studies have investigated the cellular interactome of the SARS-CoV-2 proteome including NSP12 [e.g., reference (15)]. To investigate whether the P323L substitution was potentially host adapting, we determined the cellular interactome of NSP12_P323_ and NSP12_L323_ using an EGFP trap, by expressing N- and C-terminal fusion proteins. While EGFP can be considered a larger moiety with the potential for steric hindrance, we have found this a successful combination with a viral protein to assess expression and provide targeted enrichment for mass spectrometry, particularly in the absence of specific antibodies to the viral protein (16, 19). The mass spectrometry analysis identified a potential cellular interactome of the two variants of NSP12. However, the high-confidence hits identified in this study have several in common with pre-printed studies (STRN3) (27) and components of TRiC (but not TCP-1) (28) but none in common with those previously reported for NSP12 in a published study (15). This may be due to the differing bait/capture systems that were used and/or the filtering processes. In this study, selected interactions identified by the mass spectrometry analysis were validated with both forward and reverse pulldowns coupled to western blot (Fig. 3A and B), but still with the presence of the EGFP tag. However, we were also able to demonstrate that the interactions were likely RNA independent (Fig. 3D). Several components of TRiC were identified as associating with NSP12. To our knowledge, while not described before for the coronavirus RdRp, the interaction of TCP-1 with the influenza virus PB2 subunit of the RNA polymerase has been described and shown to be involved in replication (29). The TRiC has also been shown to be involved in the replication of arenaviruses (negative sense segmented RNA viruses) (30). Pharmacological disruption of TRiC in cells infected with SARS-CoV-2 expressing either NSP12_P323_or NSP12_L323_ (through reverse genetics) resulted in a 1–2 log fold reduction in viral titer depending on the variant (Fig. 4). We note that, in general, after 24 hours post-infection, the NSP12_L323_ variant tended to grow to a slightly higher titer in ACE2-A459 cells than the NSP12_P323_ variant, with concomitant slight differences in viral protein (nucleoprotein) and RNA abundances. This is in line with a previous study from our laboratory comparing the fitness of these two variants in human and animal models as well as in culture (11). While growth differences are less than a log between the two recombinant viruses, we postulate that together with the accompanying D614G substitution in spike (e.g., reference (3)), then at an individual and population level, both 323L and 614G contribute to viruses having a greater advantage over those with P323 and D614.

Overall, we hypothesize that TRiC stabilizes the structure of NSP12 through chaperone activity, given that the abundance of an EGFP-tagged NSP12 appeared to be lower in cells exposed to the TRiC inhibitor (Fig. 4E). We cannot discount that this may be an off-target effect as TRiC ay also interact with other SARS-CoV-2 proteins critical to virus replication. However, we note that this has not been described in other interactome studies (15). Ideally, we would have isolated the activity of NSP12 from other viral proteins, for example, in the context of TRiC disruption in a mini-replicon system. However, we have not had success recapitulating mini-replicon systems described in preprinted studies.

Interestingly, we interpret the interactome data to suggest that two cellular proteins had a greater association with an EGFP-tagged NSP12_L323_ than the equivalent NSP12_P323_ variant. We note that this interactome data were quantitative but cannot be used to directly infer binding kinetics. These were STRN3, a regulatory subunit of the phosphatase, PP2Aa (23). We postulate that this is a host-adapting mutation. Similar host-adapting mutations/interactions have been described for the PB2 subunit of the influenza virus (18). Likewise, the differential activity of the influenza virus polymerase varies between mammalian and avian cells contributing toward host range (31) and mutation of the polymerase complex led to adaptation in a new species (32, 33).

Undoubtedly, the D614G substitution in the spike protein resulted in a gain in fitness that manifested as a transmission advantage in humans. We would argue that the P323L substitution in NSP12 was not just a passive change and became dominant because it featured on genomes with G614 in spike, but itself conferred a fitness advantage, and this may be due to enhanced interaction with cellular protein(s) involved in virus replication. This would suggest that SARS-CoV-2 adaptation in humans is not just associated with changes in the spike protein and supports the paradigm that other viral proteins are involved (34).

## MATERIALS AND METHODS

### Cell culture

Human Embryonic Kidney cells (HEK-293T) were maintained in DMEM with 10% FBS with no antibiotic and cells were used up to passage 30. Vero E6 cells were maintained in the same conditions. Human ACE2-A549 (hACE2-A549), a lung epithelial cell line that overexpresses human ACE2 receptor, was a kind gift from Oliver Schwartz (35). Cells were maintained in DMEM with 10% FBS and 10 μg/mL of Blasticidin (Invitrogen) to induce the overexpression of the ACE2 receptor. Passages 3–10 were used for experiments. All cell lines were tested regularly for mycoplasma contamination by PCR.

### Plasmid design

SARS-CoV-2 (Wuhan sequence) was used for the construction of plasmids in this study. The NSP12_P323_ and NSP12_L323_ sequences were codon-optimized for expression in human cells. These sequences were synthesized at GeneArt and cloned into the pEGFP-C1 and pEGFP-N1 vectors. The resulting plasmids and inserts were confirmed by sequencing (data not shown).

### Plasmid transfection

To obtain enough material for immunoprecipitation (IP), 293T cells were seeded in two 145 cm^2^ dishes at 5 × 10^6^ cells per dish 24 hours prior to calcium phosphate transfection. Cells were transfected with 25.6 μg of plasmid DNA encoding pEGFP, pEGFP-NSP12_P323_, pNSP12_P323_-EGFP, pEGFP-NSP12_L323,_ and pNSP12_L323_-EGFP. The cells were harvested 24 hours post-transfection, lysed, and immunoprecipitated using a GFP-Trap (Chromotek). Localization patterns of the fusion proteins were captured on either an ECHO revolve (low resolution) or high-resolution images on a Zeiss Cell discoverer 7 with a 50× water immersion objective and stained with DAPI to highlight the nucleus.

### EGFP co-immunoprecipitations

pEGFP, pEGFP-NSP12_P323_, pNSP12_P323_-EGFP, pEGFP-NSP12_L323_, and pNSP12_L323_-EGFP transfected cells were harvested using a cell scraper and transferred to a 50 mL tube. Cells were pelleted at 1000 × *g* for 5 min followed by three washes with 10 mL of PBS. Cell pellets were resuspended in 200 μL of lysis buffer (10 mM Tris/Cl pH 7.5; 150 mM NaCl; 0.5 mM EDTA; 0.5% NP40) supplemented with Halt Protease Inhibitor Cocktail EDTA-Free (Thermo Scientificand incubated for 30 min on ice. Lysates were transferred to 1.5 mL tubes and clarified by centrifugation at 14,000 × *g*, and the supernatants were transferred to new tubes and diluted fivefold with dilution buffer. GFP-Traps were equilibrated with ice-cold dilution buffer, and diluted cell lysate was incubated with equilibrated beads on a rotator overnight at 4°C. Beads containing proteins were washed and centrifuged at 2,500 × *g* three times for 2 min to remove non-bound proteins. Proteins were eluted from beads through heating in 1× sample buffer at 85°C for 10 min. The solution was centrifuged at 2,500 × *g* for 2 min, and the supernatant was transferred to protein low bind tubes and stored at −80°C for further analysis.

### Reverse co-immunoprecipitation

The immunoprecipitations using antibodies against CCT1, CCT5, CCT7, and STRN3 were carried out utilizing immobilized recombinant protein A/G resin (Thermo Scientific). Cell pellets were harvested and processed as described in the EGFP co-immunoprecipitation section. Cell lysates were incubated with a concentration of antibodies recommended by the supplier for 2 hours on a rotator at 4°C. Protein A/G resin (50 μL) was equilibrated with ice-cold dilution buffer, incubated overnight at 4°C on a rotator with diluted cell lysate containing the antibody, and then centrifuged at 2,500 × *g* for 2 min to remove non-bound proteins. Then, the A/G protein resin with the target protein was washed and eluted as previously described in EGFP co-immunoprecipitation.

### Protein digestion

In-gel digestion was performed as described in reference (36). Eluted proteins (20 µL, in reducing sample buffer) were run approximately 1 cm in 4%–12% NuPage gel (Invitrogen) before staining with Coomassie blue (GelCode Blue Safe Protein Stain, Fisher) for at least 1 hour, and then de-stained with ultrapure water for at least 2 hours. The entire lane length (1 mm wide) was excised and cut into smaller pieces (approximately 1 mm^3^) before de-staining with 25 mM ammonium bicarbonate/50% acetonitrile (vol/vol). Proteins were reduced for 10 min at 60°C with 10 mM dithiothreitol (Sigma) in 25 mM ammonium bicarbonate and then alkylated with 55 mM iodoacetamide (Sigma) in 50 mM ammonium bicarbonate for 30 min in the dark at room temperature. Gel pieces were washed for 15 min in 50 mM ammonium bicarbonate and then dehydrated with 100% acetonitrile. Acetonitrile was removed, and the gel plugs were rehydrated with 0.01 µg/µL proteomic grade trypsin (Thermo Scientific) in 25 mM ammonium bicarbonate. Digestion was performed overnight at 37°C. Peptides were extracted with 50% (vol/vol) acetonitrile and 0.1% TFA (vol/vol), and the extracts were reduced to dryness using a centrifugal vacuum concentrator (Eppendorf) and re-suspended in 3% (vol/vol) methanol and 0.1% (vol/vol) TFA for analysis by MS.

### NanoLC MS ESI MS/MS analysis

Liquid chromatography-mass spectrometry (LC-MS/MS) analysis was performed similar to that described by (37). Peptides were analyzed by online nanoflow LC using the Ultimate 3000 nano system (Dionex/Thermo Scientific). Samples were loaded onto a trap column (Acclaim PepMap 100, 2 cm ×75 µm inner diameter, C18, 3 µm, 100 Å) at 9 µL /min with an aqueous solution containing 0.1% (vol/vol) TFA and 2% (vol/vol) acetonitrile. After 3 min, the trap column was set in line with an analytical column (Easy-Spray PepMap RSLC 50 cm ×75 µm inner diameter, C18, 2 µm, 100 Å) fused to a silica nano-electrospray emitter (Dionex). The column was operated at a constant temperature of 35°C, and the LC system was coupled to a Q-Exactive mass spectrometer (Thermo Scientific). Chromatography was performed with a buffer system consisting of 0.1% formic acid (buffer A) and 80% acetonitrile in 0.1% formic acid (buffer B). The peptides were separated by a linear gradient of 3.8 %–50% buffer B over 30 min at a flow rate of 300 nL/min. The Q-Exactive was operated in data-dependent mode with survey scans acquired at a resolution of 70,000 at *m*/*z* 200. The scan range was 300–2000 *m*/*z*. Up to the top 10 most abundant isotope patterns with charge states from + 2 to +5 from the survey scan were selected with an isolation window of 2.0Th and fragmented by higher energy collisional dissociation with normalized collision energies of 30. The maximum ion injection times for the survey scan and the MS/MS scans were 250 and 50 ms, respectively, and the ion target value was set to 1 × 10^6^ for the survey scans and 1 × 10^4^ for the MS/MS scans. MS/MS events were acquired at a resolution of 17,500. Repetitive sequencing of peptides was minimized through dynamic exclusion of the sequenced peptides for 20 s.

### Protein identification

MS spectra data were analyzed by label-free quantification using MaxQuant software [version 1.6.17.0 (38)] and searched against the human protein database (Uniprot UP000005640_9606, May 2021) and the SARS-CoV-2 NSP12 bait protein (NCBI reference sequence: YP_009725307.1). The detected proteins were filtered to 1% false discovery rate. MaxQuant results were further processed with SAINTexpress (version 3.6.3, 39) using default settings. High confidence interactions were classified as having a SAINTexpress BFDR ≤ 0.05, and an average spectral count ≥ 2. Additional filtering to highlight unique interactors was performed using MiST in HIV-trained mode (22). Proteins with scores above 0.6 were deemed unique to that bait. Protein protein interaction (PPI) networks were generated in Cytoscape (40) with protein-protein interactions, and functional enrichment was performed using STRING (41).

### Cell viability assay

Cell viability was measured using the CellTiter-Glo Luminescent Cell Viability Assay (Promega). Cells were seeded in opaque-walled clear 96-well plates (Corning) in triplicate for each condition. The plate was equilibrated at room temperature for 30 min and 100 μL of the reagent was added. The plate was shaken at 450 rpm for 2 min and incubated for 10 min at room temperature. Luminescence was measured using the GloMax Explorer Reader (Promega) with an integration time of 0.3 s per well. All values were normalized to the control, which was set to 100%.

### siRNA knockdown

Lipofectamine (Invitrogen) (1.4 μL of RNAiMAX) was added to 23.6 μL of Opti-MEM I reduced serum media in a 1.5 μL tube. In a separate tube, the final concentration of a non-targeting siRNA or targeting siRNA was used at a concentration of 10 nM per well in a 25 μL volume of Opti-MEM I reduced serum media. siRNA was added onto the RNAiMAX and incubated for 5 min at room temperature. The incubated complex (50 μL) was added to each well, and hACE2-A549 cells in complete media in the absence of antibiotic were seeded at 0.05 × 10^6^ and incubated for 48 hours at 37°C and in 5% CO_2_. Whole cell lysates were collected and analyzed by western blot analysis to measure the knockdown efficiency.

### Recombinant viruses and infection

Two recombinant viruses were used in this study, termed SARS-CoV-2 NSP12_P323_ and SARS-CoV-2 NSP12_L323_. These viruses expressed NSP12 with either P323 or L323 and have been previously described using the “transformation-associated recombination” in yeast approach (11). As described, whole genome sequencing was used to confirm the presence of these changes. Stocks of the viruses were cultured in Vero E6 cells in DMEM containing 2% FBS and 0.05 mg/mL gentamicin and harvested 72 hours post-inoculation. Virus stocks were aliquoted and stored at −80°C. All stocks were titered by plaque assay on Vero E6 cells. Cells of ACE2-A549 were inoculated with either recombinant viruses expressing NSP12_P323_ or NSP12_L323_ for 1 hour at 37°C and in 5% CO_2_. The inoculum was removed and replaced with DMEM containing 2% FBS with no antibiotic, or DMEM containing a different dose of HSF1A for TRiC inhibition study, for 24 hours.

### Quantitative real-time PCR

RNA from cells infected with the virus was extracted with TRIzol reagent (Fisher) according to the manufacturer’s protocol. Extracted RNA was then subjected to Turbo DNase treatment (Invitrogen). RNA (200 ng) was reverse transcribed into cDNA using LunaScriptRT SuperMix (NEB) according to the manufacturer’s protocol. Generated cDNA was diluted 1:4 in nuclease-free water. Primers (42) targeting SARS-CoV-2 gRNA and N protein sgRNA were used for the quantification by RT-qPCR using iTaq Universal SYBER Green Supermix (Bio-Rad) and normalized to β-actin using 2^-ΔΔCT^.

### Western blot analysis

Cells were lysed in either RIPA buffer or IP lysis buffer (for ACE2-A549 infected cells or HEK-293T transfected cells) in the presence of a protease inhibitor cocktail (Halt Protease Inhibitor Cocktail EDTA-Free, Thermo Scientific). For ACE2-A549, 5 μg of whole cell lysate was heated at 70°C in 4× sample buffer for 10 min and then separated by 10% SDS-PAGE for 1 hour. Proteins were transferred onto the PVDF membrane. To block the membrane, 5% non-fat milk in 0.1% tris-buffered saline with tween 20 (TBST) was used for 1 hour at room temperature. The blots were then incubated with primary antibodies overnight at 4°C and secondary antibodies for 1 hour at room temperature. Blots were washed in 0.1% TBST for 5 min three times each between each antibody. The blots were imaged using a chemiluminescent reagent (ECL) with the ChemiDoc gel imaging system (Bio-Rad).

### Plaque assay

A plaque assay was used to determine the virus titer of the supernatant collected from each condition. A 10-fold dilution of each supernatant was used to inoculate ≈ 90% confluent Vero E6 cells in duplicate for 1 hour at 37°C and 5% CO_2_. The inoculum was removed and replaced with DMEM containing 2% FBS and 2% low-melting-point agarose and incubated at 37°C and 5% CO_2_ for 72 hours. Formalin (10%) was added to each well for 1 hour. Overlay containing formalin was removed, and the plates were stained with crystal violet.

### Statistical analysis

All the analyses (except the mass spectrometry data) were performed using GraphPad Prism software. One-way ANOVA test was used along with Dunnett’s multiple comparison test. The significance level in this study was set at *P*-value of less than 0.05. All error bars represent the standard deviation.

## SUPPLEMENTAL MATERIAL

Supplementary Figures jvi.00424-23-s0001.docx Fig. S1 to S7.

Supplementary tables jvi.00424-23-s0002.xlsx Interactome/mass spectrometry data.

ASM does not own the copyrights to Supplemental Material that may be linked to, or accessed through, an article. The authors have granted ASM a non-exclusive, world-wide license to publish the Supplemental Material files. Please contact the corresponding author directly for reuse.

## References

[1] Worobey M , Levy JI , Malpica Serrano LM , Crits-Christoph A , Pekar JE , Goldstein SA , Rasmussen AL , Kraemer MU , Newman C , Koopmans MPG , Suchard MA , Wertheim JO , Lemey P , Robertson DL , Garry RF , Holmes EC , Rambaut A , Andersen KG . 2022. The huanan market was the epicenter of SARS-CoV-2 emergence. Zenodo. doi:10.5281/zenodo.6299600 PMC9348750 35881010

[2] Pekar JE , Magee A , Moshiri N , Izhikevich K , Havens JL , Gangavarapu K , Serrano LMM , Crits-Christoph A , Matteson NL , Zeller M , et al. . 2022. SARS-CoV-2 emergence very likely resulted from at least two zoonotic events. Zenodo. doi:10.5281/zenodo.6291628

[3] Plante JA , Liu Y , Liu J , Xia H , Johnson BA , Lokugamage KG , Zhang X , Muruato AE , Zou J , Fontes-Garfias CR , Mirchandani D , Scharton D , Bilello JP , Ku Z , An Z , Kalveram B , Freiberg AN , Menachery VD , Xie X , Plante KS , Weaver SC , Shi PY . 2021. Spike mutation D614G alters SARS-CoV-2 fitness. Nature 592:116–121. doi:10.1038/s41586-020-2895-3 33106671 PMC8158177

[4] Zhang L , Jackson CB , Mou H , Ojha A , Peng H , Quinlan BD , Rangarajan ES , Pan A , Vanderheiden A , Suthar MS , Li W , Izard T , Rader C , Farzan M , Choe H . 2020. SARS-CoV-2 spike-protein D614G mutation increases virion spike density and infectivity. Nat Commun 11:6013. doi:10.1038/s41467-020-19808-4 33243994 PMC7693302

[5] Worobey M , Pekar J , Larsen BB , Nelson MI , Hill V , Joy JB , Rambaut A , Suchard MA , Wertheim JO , Lemey P . 2020. The emergence of SARS-CoV-2 in Europe and north America. Science 370:564–570. doi:10.1126/science.abc8169 32912998 PMC7810038

[6] Volz E , Mishra S , Chand M , Barrett JC , Johnson R , Geidelberg L , Hinsley WR , Laydon DJ , Dabrera G , O’Toole Á , et al. . 2021. Assessing transmissibility of SARS-CoV-2 lineage B.1.1.7 in England. Nature 593:266–269. doi:10.1038/s41586-021-03470-x 33767447

[7] Mlcochova P , Kemp SA , Dhar MS , Papa G , Meng B , Ferreira I , Datir R , Collier DA , Albecka A , Singh S , et al. . 2021. SARS-CoV-2 B.1.617.2 delta variant replication and immune evasion. Nature 599:114–119. doi:10.1038/s41586-021-03944-y 34488225 PMC8566220

[8] Andrews N , Stowe J , Kirsebom F , Toffa S , Rickeard T , Gallagher E , Gower C , Kall M , Groves N , O’Connell A-M , et al. . 2022. Covid-19 vaccine effectiveness against the omicron (B.1.1.529) variant. N Engl J Med 386:1532–1546. doi:10.1056/NEJMoa2119451 35249272 PMC8908811

[9] Peacock TP , Penrice-Randal R , Hiscox JA , Barclay WS . 2021. SARS-CoV-2 one year on: evidence for ongoing viral adaptation. J Gen Virol 102:001584. doi:10.1099/jgv.0.001584 33855951 PMC8290271

[10] Ilmjärv S , Abdul F , Acosta-Gutiérrez S , Estarellas C , Galdadas I , Casimir M , Alessandrini M , Gervasio FL , Krause K-H . 2021. Concurrent mutations in RNA-dependent RNA polymerase and spike protein emerged as the epidemiologically most successful SARS-CoV-2 variant. Sci Rep 11:13705. doi:10.1038/s41598-021-91662-w 34210996 PMC8249556

[11] Goldswain H , Dong X , Penrice-Randal R , Alruwaili M , Shawli GT , Prince T , Williamson MK , Raghwani J , Randle N , Jones B , Donovan-Banfield I , et al. . 2023. The P323L substitution in the SARS-CoV-2 polymerase (NSP12) confers a selective advantage during infection. Genome Biol 24:47. doi:10.1186/s13059-023-02881-5 36915185 PMC10009825

[12] Wang Q , Wu J , Wang H , Gao Y , Liu Q , Mu A , Ji W , Yan L , Zhu Y , Zhu C , Fang X , Yang X , Huang Y , Gao H , Liu F , Ge J , Sun Q , Yang X , Xu W , Liu Z , Yang H , Lou Z , Jiang B , Guddat LW , Gong P , Rao Z . 2020. Structural basis for RNA replication by the SARS-CoV-2 polymerase. Cell 182:417–428. doi:10.1016/j.cell.2020.05.034 32526208 PMC7242921

[13] Kirchdoerfer RN , Ward AB . 2019. Structure of the SARS-CoV nsp12 polymerase bound to nsp7 and nsp8 co-factors. Nat Commun 10:2342. doi:10.1038/s41467-019-10280-3 31138817 PMC6538669

[14] Wolff G , Limpens RWAL , Zevenhoven-Dobbe JC , Laugks U , Zheng S , de Jong AWM , Koning RI , Agard DA , Grünewald K , Koster AJ , Snijder EJ , Bárcena M . 2020. A molecular pore spans the double membrane of the coronavirus replication organelle. Science 369:1395–1398. doi:10.1126/science.abd3629 32763915 PMC7665310

[15] Gordon DE , Jang GM , Bouhaddou M , Xu J , Obernier K , White KM , O’Meara MJ , Rezelj VV , Guo JZ , Swaney DL , et al. . 2020. A SARS-CoV-2 protein interaction map reveals targets for drug repurposing. Nature 583:459–468. doi:10.1038/s41586-020-2286-9 32353859 PMC7431030

[16] Munday DC , Wu W , Smith N , Fix J , Noton SL , Galloux M , Touzelet O , Armstrong SD , Dawson JM , Aljabr W , Easton AJ , Rameix-Welti M-A , de Oliveira AP , Simabuco FM , Ventura AM , Hughes DJ , Barr JN , Fearns R , Digard P , Eléouët J-F , Hiscox JA . 2015. Interactome analysis of the human respiratory syncytial virus RNA polymerase complex identifies protein chaperones as important cofactors that promote L-protein stability and RNA synthesis. J Virol 89:917–930. doi:10.1128/JVI.01783-14 25355874 PMC4300676

[17] Taguwa S , Maringer K , Li X , Bernal-Rubio D , Rauch JN , Gestwicki JE , Andino R , Fernandez-Sesma A , Frydman J . 2015. Defining Hsp70 subnetworks in dengue virus replication reveals key vulnerability in flavivirus infection. Cell 163:1108–1123. doi:10.1016/j.cell.2015.10.046 26582131 PMC4869517

[18] Camacho-Zarco AR , Kalayil S , Maurin D , Salvi N , Delaforge E , Milles S , Jensen MR , Hart DJ , Cusack S , Blackledge M . 2020. Molecular basis of host-adaptation interactions between influenza virus polymerase PB2 subunit and ANP32A. Nat Commun 11:3656. doi:10.1038/s41467-020-17407-x 32694517 PMC7374565

[19] Dong X , Munoz-Basagoiti J , Rickett NY , Pollakis G , Paxton WA , Günther S , Kerber R , Ng LFP , Elmore MJ , Magassouba N , Carroll MW , Matthews DA , Hiscox JA . 2020. Variation around the dominant viral genome sequence contributes to viral load and outcome in patients with ebola virus disease. Genome Biol 21:238. doi:10.1186/s13059-020-02148-3 32894206 PMC7475720

[20] Emmott E , Munday D , Bickerton E , Britton P , Rodgers MA , Whitehouse A , Zhou EM , Hiscox JA . 2013. The cellular interactome of the coronavirus infectious bronchitis virus nucleocapsid protein and functional implications for virus biology. J Virol 87:9486–9500. doi:10.1128/JVI.00321-13 23637410 PMC3754094

[21] Choi H , Larsen B , Lin ZY , Breitkreutz A , Mellacheruvu D , Fermin D , Qin ZS , Tyers M , Gingras AC , Nesvizhskii AI . 2011. SAINT: probabilistic scoring of affinity purification-mass spectrometry data. Nat Methods 8:70–73. doi:10.1038/nmeth.1541 21131968 PMC3064265

[22] Verschueren E , Von Dollen J , Cimermancic P , Gulbahce N , Sali A , Krogan NJ . 2015. Scoring large-scale affinity purification mass spectrometry datasets with MiST. Curr Protoc Bioinformatics 49:8. doi:10.1002/0471250953.bi0819s49 PMC4378866 25754993

[23] Tang Y , Fang G , Guo F , Zhang H , Chen X , An L , Chen M , Zhou L , Wang W , Ye T , Zhou L , Nie P , Yu H , Lin M , Zhao Y , Lin X , Yuan Z , Jiao S , Zhou Z . 2020. Selective inhibition of STRN3-containing PP2A phosphatase restores hippo tumor-suppressor activity in gastric cancer. Cancer Cell 38:115–128. doi:10.1016/j.ccell.2020.05.019 32589942

[24] Neef DW , Jaeger AM , Gomez-Pastor R , Willmund F , Frydman J , Thiele DJ . 2014. A direct regulatory interaction between chaperonin TRIC and stress-responsive transcription factor HSF1. Cell Rep 9:955–966. doi:10.1016/j.celrep.2014.09.056 25437552 PMC4488849

[25] Zhang Y , Huang K , Xie D , Lau JY , Shen W , Li P , Wang D , Zou Z , Shi S , Ren H , Wang Y , Mao Y , Jin M , Kudla G , Zhao Z . 2021. In vivo structure and dynamics of the SARS-CoV-2 RNA genome. Nat Commun 12:5695. doi:10.1038/s41467-021-25999-1 34584097 PMC8478942

[26] Periwal N , Rathod SB , Sarma S , Johar GS , Jain A , Barnwal RP , Srivastava KR , Kaur B , Arora P , Sood V . 2022. Time series analysis of SARS-CoV-2 genomes and correlations among highly prevalent mutations. Microbiol Spectr 10:e0121922. doi:10.1128/spectrum.01219-22 36069583 PMC9603882

[27] Bouhaddou M , Reuschl A-K , Polacco BJ , Thorne LG , Ummadi MR , Ye C , Rosales R , Pelin A , Batra J , Jang GM , et al. . 2022. Global landscape of the host response to SARS-CoV-2 variants reveals viral evolutionary trajectories. bioRxiv. doi:10.1101/2022.10.19.512927:2022.10.19.512927

[28] Laurent EMN , Sofianatos Y , Komarova A , Gimeno J-P , Tehrani PS , Kim D-K , Abdouni H , Demeret C , Jacob Y , Coyaud E , et al. . Global BioID-based SARS-CoV-2 proteins proximal interactome unveils novel ties between viral polypeptides and host factors involved in multiple COVID19-associated mechanisms bioRxiv. doi:10.1101/2020.08.28.272955:2020.08.28.272955

[29] Fislová T , Thomas B , Graef KM , Fodor E . 2010. Association of the influenza virus RNA polymerase subunit PB2 with the host chaperonin CCT. J Virol 84:8691–8699. doi:10.1128/JVI.00813-10 20573828 PMC2919027

[30] Sakabe S , Witwit H , Khafaji R , Cubitt B , de la Torre JC . 2023. Chaperonin TRiC/CCT participates in mammarenavirus multiplication in human cells via interaction with the viral Nucleoprotein. J Virol 97:e0168822. doi:10.1128/jvi.01688-22 36656012 PMC9973018

[31] Gabriel G , Abram M , Keiner B , Wagner R , Klenk HD , Stech J . 2007. Differential polymerase activity in avian and mammalian cells determines host range of influenza virus. J Virol 81:9601–9604. doi:10.1128/JVI.00666-07 17567688 PMC1951401

[32] Gabriel G , Dauber B , Wolff T , Planz O , Klenk HD , Stech J . 2005. The viral polymerase mediates adaptation of an avian influenza virus to a mammalian host. Proc Natl Acad Sci U S A 102:18590–18595. doi:10.1073/pnas.0507415102 16339318 PMC1317936

[33] Taft AS , Ozawa M , Fitch A , Depasse JV , Halfmann PJ , Hill-Batorski L , Hatta M , Friedrich TC , Lopes TJS , Maher EA , Ghedin E , Macken CA , Neumann G , Kawaoka Y . 2015. Identification of mammalian-adapting mutations in the polymerase complex of an avian H5N1 influenza virus. Nat Commun 6:7491. doi:10.1038/ncomms8491 26082035 PMC4557292

[34] Thorne LG , Bouhaddou M , Reuschl A-K , Zuliani-Alvarez L , Polacco B , Pelin A , Batra J , Whelan MVX , Hosmillo M , Fossati A , Ragazzini R , Jungreis I , Ummadi M , Rojc A , Turner J , Bischof ML , Obernier K , Braberg H , Soucheray M , Richards A , Chen K-H , Harjai B , Memon D , Hiatt J , Rosales R , McGovern BL , Jahun A , Fabius JM , White K , Goodfellow IG , Takeuchi Y , Bonfanti P , Shokat K , Jura N , Verba K , Noursadeghi M , Beltrao P , Kellis M , Swaney DL , García-Sastre A , Jolly C , Towers GJ , Krogan NJ . 2022. Evolution of enhanced innate immune evasion by SARS-CoV-2. Nature 604:487–495. doi:10.1038/s41586-022-04653-w PMC8850198 34942634

[35] Buchrieser J , Dufloo J , Hubert M , Monel B , Planas D , Rajah MM , Planchais C , Porrot F , Guivel-Benhassine F , Van der Werf S , Casartelli N , Mouquet H , Bruel T , Schwartz O . 2020. Syncytia formation by SARS-CoV-2-infected cells. EMBO J 39:e106267. doi:10.15252/embj.2020106267 33051876 PMC7646020

[36] Shevchenko A , Tomas H , Havlis J , Olsen JV , Mann M . 2006. In-gel digestion for mass spectrometric characterization of proteins and proteomes. Nat Protoc 1:2856–2860. doi:10.1038/nprot.2006.468 17406544

[37] Aljabr W , Armstrong S , Rickett NY , Pollakis G , Touzelet O , Cloutman-Green E , Matthews DA , Hiscox JA . 2019. High resolution analysis of respiratory syncytial virus infection in vivo. Viruses 11:926. doi:10.3390/v11100926 31658630 PMC6832471

[38] Cox J , Hein MY , Luber CA , Paron I , Nagaraj N , Mann M . 2014. Accurate proteome-wide label-free quantification by delayed normalization and maximal peptide ratio extraction, termed MaxLFQ. Mol Cell Proteomics 13:2513–2526. doi:10.1074/mcp.M113.031591 24942700 PMC4159666

[39] Teo G , Liu G , Zhang J , Nesvizhskii AI , Gingras A-C , Choi H . 2014. SAINTexpress: improvements and additional features in significance analysis of interactome software. J Proteomics 100:37–43. doi:10.1016/j.jprot.2013.10.023 24513533 PMC4102138

[40] Shannon P , Markiel A , Ozier O , Baliga NS , Wang JT , Ramage D , Amin N , Schwikowski B , Ideker T . 2003. Cytoscape: a software environment for integrated models of biomolecular interaction networks. Genome Res 13:2498–2504. doi:10.1101/gr.1239303 14597658 PMC403769

[41] Doncheva NT , Morris JH , Gorodkin J , Jensen LJ . 2019. Cytoscape stringapp: network analysis and visualization of proteomics data. J Proteome Res 18:623–632. doi:10.1021/acs.jproteome.8b00702 30450911 PMC6800166

[42] White KM , Rosales R , Yildiz S , Kehrer T , Miorin L , Moreno E , Jangra S , Uccellini MB , Rathnasinghe R , Coughlan L , Martinez-Romero C , Batra J , Rojc A , Bouhaddou M , Fabius JM , Obernier K , Dejosez M , Guillén MJ , Losada A , Avilés P , Schotsaert M , Zwaka T , Vignuzzi M , Shokat KM , Krogan NJ , García-Sastre A . 2021. Plitidepsin has potent preclinical efficacy against SARS-CoV-2 by targeting the host protein eEF1A. Science 371:926–931. doi:10.1126/science.abf4058 33495306 PMC7963220

