# Supplementary material for: SARS-CoV-2 NSP12 associates with TRiC and the P323L substitution acts as a host adaption
Source: J Virol. 2023 Nov 6;97(11):e00424-23. doi: 10.1128/jvi.00424-23 (PMC10688337; doi:10.1128/jvi.00424-23)
Supplement: Supplementary Figures — Fig. S1 to S7. [file jvi.00424-23-s0001.docx]

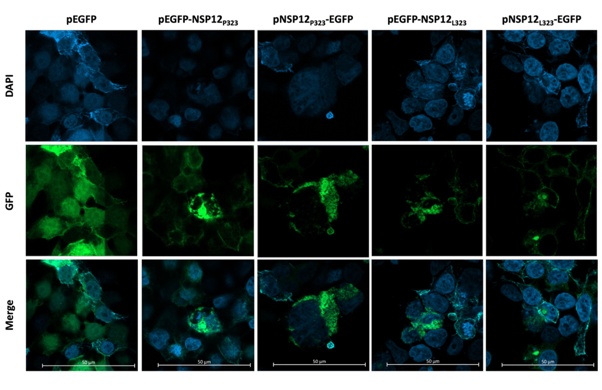


**Supplementary Figure 1. Super high resolution imaging analysis of the expression of EGFP, and EGFP tagged NSP12_P323_, and NSP12_L323_ at the C- and N- terminus – as indicated.** DAPI was used to stain the nucleus and both the GFP and merge channels are shown. Scale is indicated at the lower part of the image.


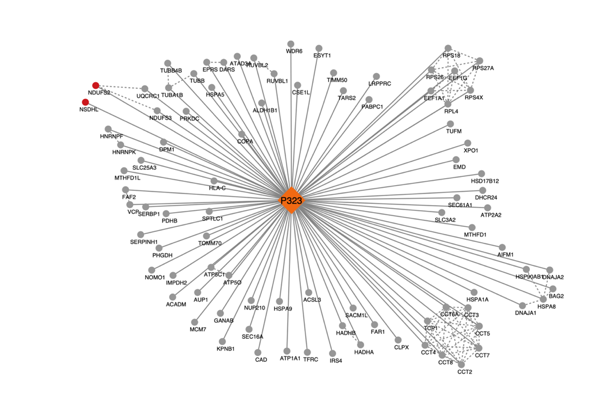


**Supplementary Figure 2. Host Protein Interactome with EGFP-NSP12_P323_.** The putative protein-protein interaction network of NSP12_P323_ with an n-terminal GFP tag was obtained via affinity pulldown mass spectrometry (AP-MS). PPI networks were created using cytoscape and STRING. The grey nodes denote high confidence interacting proteins (SAINTexpress BFDR score ≤ 0.05). The red nodes denote proteins that are unique to this NSP12 construct (BFDR score ≤0.05, MiST score ≥0.6). Solid lines represent putative interaction with the bait protein. Dashed lines represent previously known associations obtained via STRING. Proteins with enhanced association with EGFP-NSP12_P323_ include NSDHL (Sterol-4-alpha-carboxylate 3-dehydrogenase) which is involved in sterol synthesis and NDUFS2 (NADH dehydrogenase [ubiquinone] iron-sulfur protein 2), a mitochondrial protein subunit in Respiratory complex I. Protein clusters involved with translation (RPS27A, RPL4, EEF1G, EEF1A1, RPS7, RPL3, RPS26, RPS4X, RPL11, RPS18, RPL10, RPS19) and protein folding (TRiC complex, CCT-CCT8, TCP1) are highly enriched.

**Supplementary Figure 3. Host Protein Interactome with EGFP-NSP12_L323_.** The putative protein-protein interaction network of NSP12_L323_ with an n-terminal GFP tag was obtained via affinity pulldown mass spectrometry (AP-MS). PPI networks were created using cytoscape and STRING. The grey nodes denote high confidence interacting proteins (SAINTexpress BFDR score ≤ 0.05). The red nodes denote proteins that are unique to this NSP12 construct (BFDR score ≤0.05, MiST score ≥0.6). Solid lines represent putative interaction with the bait protein. Dashed lines represent previously known associations obtained via STRING. Proteins with enhanced association with EGFP-NSP12_L323_ include EPPK1 (Epiplakin), which has roles in reorganization of the cytoskeleton and cell proliferation and STRN3 (striatin-3) and PPP2R1A (Serine/threonine-protein phosphatase 2A) which are part of the PP2A phosphatase family. Protein clusters involved with translation (RPS27A, RPL4, EEF1G, EEF1A1, RPS7, RPL3, RPS26, RPS4X, RPL11, RPS18, RPL10, RPS19) and protein folding (TRiC complex, CCT-CCT8, TCP1) are highly enriched.


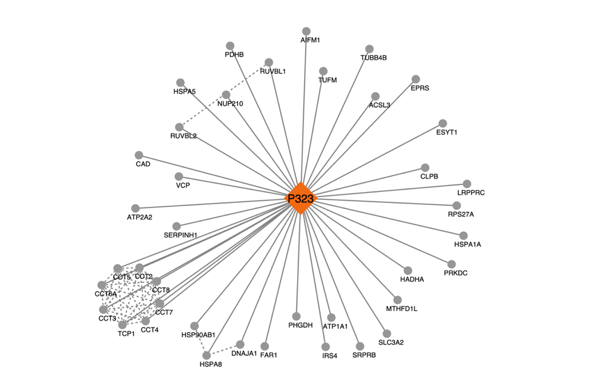


**Supplementary Figure 4. Host Protein Interactome with NSP12_P323_-EGFP.** The putative protein-protein interaction network of NSP12_P323_ with an c-terminal GFP tag was obtained via affinity pulldown mass spectrometry (AP-MS). PPI networks were created using cytoscape and STRING. The grey nodes denote high confidence interacting proteins (SAINTexpress BFDR score ≤ 0.05). The red nodes denote proteins that are unique to this NSP12 construct (BFDR score ≤0.05, MiST score ≥0.6). Solid lines represent putative interaction with the bait protein. Dashed lines represent previously known associations obtained via STRING. There are no proteins with enhanced association with NSP12_P323_. Protein cluster involved with protein folding (TRiC complex, CCT-CCT8, TCP1) is highly enriched.


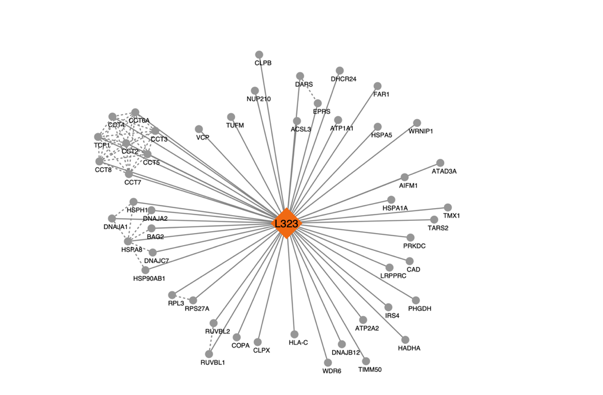


**Supplementary Figure 5. Host Protein Interactome with NSP12_L323_-EGFP.** The putative protein-protein interaction network of NSP12_L323_ with an c-terminal GFP tag was obtained via affinity pulldown mass spectrometry (AP-MS). PPI networks were created using cytoscape and STRING. The grey nodes denote high confidence interacting proteins (SAINTexpress BFDR score ≤ 0.05). The red nodes denote proteins that are unique to this NSP12 construct (BFDR score ≤0.05, MiST score ≥0.6). Solid lines represent putative interaction with the bait protein. Dashed lines represent previously known associations obtained via STRING. There are no proteins with enhanced association with NSP12_L323_. Protein cluster involved with protein folding (TRiC complex, CCT-CCT8, TCP1) is highly enriched.


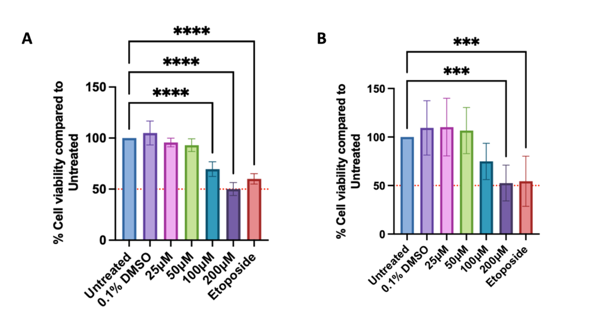


**Supplementary Figure 6. Cell viability assay (CellTiter Glo assay) of ACE2-A549 and HEK-293T cells treated with the TRiC/CCT complex inhibitor, HSF1A.** (A) ACE2-A549 or (B) HEK-293T cells were treated with a range of HSF1A concentrations from 25 to 200 μM for 28 h. Untreated cells were used as a control; 60 µM of Etoposide was used as a positive control for the induction of cell death. The luminescence for untreated cells was normalised to 100%, then % cell viability for different HSF1A doses were calculated relative to this. The dashed red line indicates the GI50. *** p<0.001 and **** p<0.0001 by one-way ANOVA with Dunnett’s multiple comparison test. Data shown represent the mean of three independent experiments. All error bars represent the standard deviation.

**
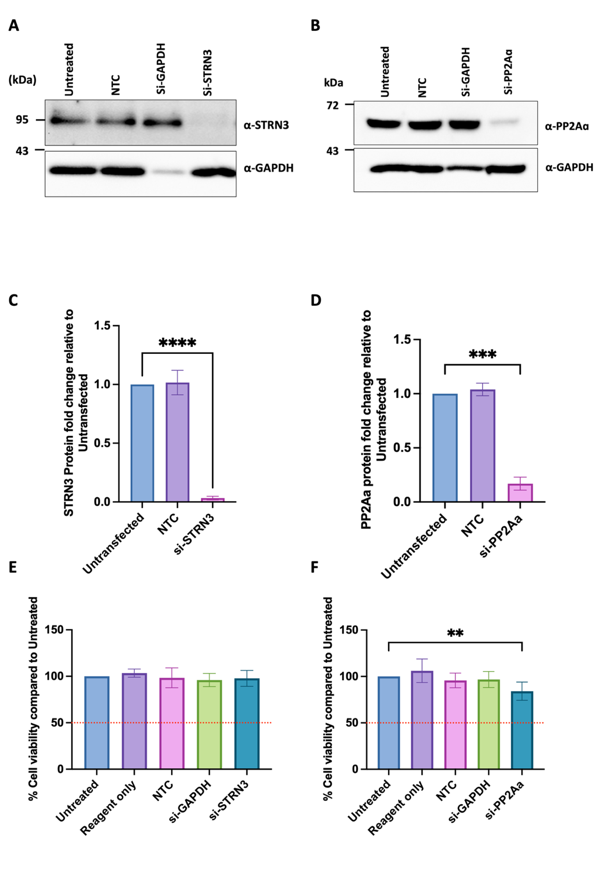
**

**Supplementary Figure 7. STRN3 and PP2Aa siRNA knockdown efficiency**. (A) ACE2-A549 cells were reverse-transfected with 10 nM of either non-targeting siRNA control (NTC) alongside si-GAPDH (to monitor knockdown efficiency) and si-STRN3. After 48 h, whole cell lysates were collected and evaluated for the expression levels of STRN3 by western blot analysis. (B) ACE2-A549 cells were reverse-transfected with 10 nM of either non-targeting siRNA control (NTC) alongside si-GAPDH (to monitor knockdown efficiency) and si-PP2Aa. After 48 h, whole cell lysates were collected and evaluated for the expression levels of PP2Aa by western blot analysis. ImageJ quantification of (C) STRN3 or (D) PP2Aa expressions and normalised to the internal control GAPDH. ACE2-A549 cells were reverse-transfected in 96 well plate format with 10 nM of either non-targeting siRNA control (NTC) alongside si-GAPDH (E) si-STRN3, or (F) si-PP2Aa for 48 h. the mean luminescence for untreated cells was normalised to 100%, and then % cell viability for different conditions were calculated relative to this. Data shown represent the mean of three independent experiments. All error bars represent the standard deviation.
